# Shared Decision-Making: An Autoethnography About Service User Perspectives in Making Choices About Mental Health Care and Treatment

**DOI:** 10.3389/fpsyt.2021.637560

**Published:** 2021-03-10

**Authors:** Joanna Fox

**Affiliations:** School of Education and Social Care, Anglia Ruskin University, Cambridge, United Kingdom

**Keywords:** medication choices, autoethnography, service user perspective, prescribers, well-being

## Abstract

Shared decision-making (SDM) between mental health medication prescribers and service users is a central pillar in the recovery approach, because it supports people experiencing mental ill-health to explore their care and treatment options to promote their well-being and to enable clinicians to gain knowledge of the choices the service user prefers. SDM is receiving increasing recognition both in the delivery of physical and mental health services; and as such, is of significance to current practice. As an expert-by-experience with over 30 years of receiving mental health treatment, I have made many choices about taking medication and accessing other forms of support. The experiences of SDM have been variable over my career as a service user: both encounters when I have felt utterly disempowered and interactions when I have led decision-making process based on my expertise-by-experience. In this article, I recount two experiences of exploring care and treatment options: firstly, a discharge planning meeting; and secondly, the choice to take medication over the long-term, despite the side effects. The article will explore both opportunities and barriers to effective shared decision-making, as well as skills and processes to facilitate this approach. The need to balance power between service users and professionals in this interaction is highlighted, including the need to respect expertise built on lived experience, alongside that of clinical expertise. This narrative is framed within an autoethnographic approach which allows me to contextualize my personal experiences in the wider environment of mental health care and support.

## Introduction

Recovery is an aspirational practice at the center of mental health service delivery in the UK today (1, 2) and underpins the implementation of services for people experiencing complex psychosis (3). Recovery is a process which supports a person with lived experience of mental ill-health to self-manage their condition putting them at the center of decision-making about their lives (4). Using the acronym, CHIME, the essential elements of this approach are conveyed (5): recovery is perceived as a unique journey which requires Connectedness, Hope and optimism about the future, the creation of Identity, Meaning in life and the need for Empowerment. Recovery promotes the development of agency and autonomy in the lives of service users (4); thus, the process of shared decision-making (SDM) in choices about mental health interventions enables people who use services to co-produce recovery in partnership with the practitioner (6). This article will explore my experiences of decision-making processes in two professional encounters as a user of mental health services for over 30 years, enabling me to illuminate this approach from my perspective as both an expert-by-experience and a social work academic. This narrative is framed within an autoethnographic approach (7, 8), which allows me to contextualize my personal experiences in the wider environment of mental health support. Moreover, it provides me with the opportunity to investigate what makes effective SDM in the process of clinical interventions from my hybrid standpoint as both a social care professional and a service user expert.

Shared decision-making (9) is defined as “a process in which decisions are made in a collaborative way, where, trustworthy information is provided in accessible formats about a set of options, typically in situations where the concerns, personal circumstances, and contexts of patients and their families play a major role in decisions.” SDM lies along a continuum of forms of decision-making in health and social care settings which range from paternalistic to informed choice approaches (10, 11). The advantages of SDM include increased therapeutic alliance, enhanced shared knowledge and understanding of key intervention issues, saving time in review meetings, and an increased commitment to implementing decisions jointly taken (6). Moreover, in a systematic review and meta-analysis of the evidence, collaborative decision-making around psychiatric treatment (12), in a process that considers patient preferences and values, is likely to help people receiving treatment for psychosis experience greater empowerment and reduced coercion in relation to their care. Moreover, in a study of implementation of SDM in youth early intervention services (13), family caregivers were involved in decision-making and it was posited that involvement should be negotiated on an individual basis; however, all caregivers should be supported with information about mental ill-health and treatment options.

Since 2012, UK guidance has stipulated that processes of SDM should promote choice and the development of agency for people who use mental health services (14). SDM is seen at the forefront of moves toward personalized care which “means people have choice and control over the way their care is planned and delivered, based on ‘what matters' to them and their individual strengths and needs” [(15): 3]. Moreover, personalized care is at the center of the development and delivery of health and social care in England and Wales (15); and, also, in mental health care (1, 2).

Despite this stipulation, the implementation of SDM in the care of people who use mental health care (15) in mental health policy in England (14, 15) is tempered with the need to manage risk and to ensure the safeguarding of vulnerable people. Thus, the balance between care and control in the delivery of mental health services is located between the duty to protect life under the Human Rights Act, article 2 (16), and a duty to preserve and promote choice, dignity, and freedom (17). These two poles of care provision exist at different ends of a continuum, given that professionals aim at balancing the need for care and control. Thus, SDM is a framework which is controversial for many professionals (18) making its implementation challenging for both professionals and service users (19). Additionally, its emphasis on the importance of acknowledging the value of experiential knowledge in the therapeutic alliance between the service user and the practitioner can also be demanding because it generates a new relationship between these two parties (6). It requires a shift in the behavior and attitudes of both participants in recognizing each other's expertise in this interaction. Moreover, the value of expertise-by-experience is further highlighted in an Australian study, which implemented peer support (individuals with lived experience helping other consumers) in shared decision-making processes in youth early intervention settings (20); this study emphasizes the place of experiential knowledge in SDM.

In the next section, I reflect on my experiences of decision-making in mental health management as I recount two encounters of mental health intervention framed within an autoethnographic approach. These interventions are discussed to enable an understanding of the effectiveness of SDM from my standpoint, as both an expert-by-experience and a social work academic. The reflections thus serve as a *springboard* to highlight the processes of SDM and to enable further exploration of the nature of decision-making in mental health care from the perspective of an expert-by-experience.

### Autoethnography: A Process of Reflection to Illuminate My Response to SDM

Autoethnography has been used widely in health and social care research, education, and practice (20, 21). Autoethnography is employed in this article to reflect on my experiences of SDM through a process of writing, and to position them in the wider social and political environment (7). Reflective practice has a long tradition in the helping professions as a method to develop both personal understanding of the lived experiences of service users and carers and of innovation in practice (22), therefore, autoethnography is appropriate to this article. Autoethnographic writing (7) requires the researcher to pay careful attention to both the *epistemic* (claims to knowledge) and the *aesthetic* (practices of imaginative, creative, and artistic craft) characteristics of their texts as they seek to convey the meaning of their individual experiences and communicate their significance to the wider community of practice.

Autoethnography strives for social justice (7) and promotes moral and ethical debate through the process of reflexivity (8). My accounts were analyzed by using thematic data analysis (23) and themes commensurate with the literature were identified and integrated into the discussion. Thematic analysis involves a six-phase process (23); however, it is often flexible, encompassing an approach that can be both “inductive” and “data-driven.” Thus, initially, themes were identified inductively as I read and re-read the reflections that *described* the experiences expressed in the accounts; then themes were identified which were commensurate with the published literature. Furthermore, different sources of evidence are used alongside my reports to explicitly link concepts from the literature to my narrations. Thus, both the process of reflexivity (22) as a service user and academic (24) and the narratives in the article facilitated the connection of “the autobiographical and personal to the cultural, social, and political” (25).

Ethical challenges may arise when using such a personally revealing research process as autoethnography and writing about such intimate experiences. This situation is explored by Goldberg et al. (26) who illuminate the experiences of a mental health practitioner who also became a hospital inpatient. Goldberg et al. (26) discuss the need to manage both personal and professional boundaries and to consider these needs carefully in relation to the working and professional environment; an issue highlighted in the context of social work (27).

As an expert-by-experience (24) I choose to use my lived experiences of mental distress to effect positive change in mental health care (28), but, of necessity, I have learnt to separate personal emotions from the professional domain. I communicate my encounters with services by *objectifying* the substance of these experiences to explore them as *empirical data*, rather than as occurrences full of emotional content. This division allows me to disconnect the personal from the professional and maintain a divide between these two domains. However, at times, reliving my past experiences may impact on my sense of mental well-being, and I find it necessary to pause the process of writing to give myself a break (21).

### My Experiences of SDM

In this section I recount two experiences of decision-making in clinical care: one at the start of my mental health career and one more recent experience. This illuminates an understanding of this topic from my perspective. I have taken mental health medication for over 30 years and reflect on how my own expertise and relationship with professionals has adapted and changed. I describe, firstly, one memory of early involvement in decision-making following my first stay in hospital, and, secondly, a more recent experience of trying a different mental health medication.

The first situation occurred when I was attending a planning discharge meeting whilst emerging from my first episode of acute psychosis. This was my first experience of being in hospital and of mental distress. On discussing when I would be discharged, I entered a room with more than 15 people and remember nothing more of that meeting than the number of faces staring back at me. This encounter remains in my memories after over 30 years of care, I remember there being no support, no explanation of the meeting, and no discussion of the outcome. It is a single and clear memory, with little embellishment. There was no understanding of the enormity of this experience or of the sense of fear and disempowerment I experienced; this encounter was very far from the ideal of involvement in decision-making and evidenced power being situated completely in the hands of professionals. It may have been helpful if the purpose of the meeting had been discussed in advance; if I had been informed of what to expect from the meeting; and if I could have been accompanied by an advocate or person I had got to know on the ward.

My second account relates to an experience within the last 2 years. I went through a period of excessive weight gain. I met with the psychiatrist who I have known for over 30 years. He has now semi-retired from the NHS and I chose to see him privately; otherwise, I would not be able to access mental health support in a timely way. The medication I take increases my appetite and makes me crave foods; other side effects include: an increased propensity to develop diabetes, global sedation, cloudy thought patterns, reduced libido. I wanted to explore changing medication. This drug supports me to manage anxiety by evening out the extremes of emotion, although, one disadvantage of this medication, is, I believe that it has suppressed my natural emotional responses. This is a side effect which I accept as a pay-off for managing anxiety. The psychiatrist explored my concerns about weight gain and committed to investigate some alternative treatments.

When we next met, the psychiatrist recommended some options, and I chose to take a new medication. I found it to be effective in reducing my appetite, but less effective at containing my anxiety and other feelings of paranoia. I tried the change for 2 months and then, in consultation with my psychiatrist, returned to the medication that I know works best for me; albeit I immediately gained the weight I had lost. I had to decide between taking a medication that enabled me to manage my mental ill-health and increase weight, and to take a less effective medication that did not cause weight gain. This was a decision driven by expediency as I needed to function effectively in both my personal and professional life.

These encounters highlight two specific themes that are central to the practice of SDM in mental health care: the impact that a service user's incapacity, lack of insight and acute distress can have when negotiating clinical interventions in the context of SDM; and the change in decision-making processes when the service user becomes a self- acknowledged expert-by-experience, as well as being recognized as such by practitioners.

## Discussion

The first encounter occurred 30 years ago at an inpatient discharge planning meeting following my first episode of acute psychosis. The second was a more recent encounter when I chose to see a psychiatrist as an outpatient to discuss my medication and treatment options, where I subsequently felt this experience was an example of effective SDM. The first encounter occurred at the beginning of my mental health journey, long before there were any guidelines on SDM. I would not have considered myself an expert-by-experience at the time of the first encounter; I had no comprehension of what mental illness symptoms were and had not heard of the terminology of psychosis. In the second encounter, initiated by myself, I had decades of first-hand experience with mental health practice, and was informed about the side effects of the various suitable medications for my condition, hence at that point, considered myself as an expert-by-experience. In the first encounter, the psychiatrist didn't know me, nor was I able to understand my condition or to understand what was happening to me. I remember little being discussed with me about psychosis or little information provided to me. This was a barrier to SDM in its simplest format.

These two accounts demonstrate how decisions are made in mental health care based on different kinds of expertise: the former presents an episode of an uninformed and under-involved patient, subjected to the power of the professionals, having decisions made for her; whilst the latter episode exemplifies an interaction of shared power as decisions are made based on both experiential and clinical wisdom. This second example reflects a process of SDM, because at its core is a shared agreement and shared enterprise between the service user and the professional to share risk (6). This requires the practitioner to give up some of their power and enter a more equal relationship with the service user and be open to the service user perspective (18).

My experiences of recent care have been underpinned by my expertise-by-experience and based on my own expert knowledge of my condition.

To promote SDM, in my experience, effective communication is central to interactions between the service user and the professional (19). Three elements have been identified as key to effective SDM (29): knowing the client; awareness of the practitioner; and the therapeutic relationship. The client needs to want to be involved in SDM and be trained how to engage in it, and the professional should be aware of SDM techniques and should place the therapeutic alliance with the patient at the center of the interaction, offering clear information and actively listening to the client's viewpoints. In support of this, it has been found that establishing effective relationships based on a person-centered and user participation model are more important in decision-making than following an established pathway with little consultation (30). Additionally, the three-talk model of SDM (9) highlights the need for active listening between the parties leading to discussion of possible options for the service user to make when facing a decision.

From my accounts, these elements are essential to effective SDM. Responding to my needs, clarifying information, and respecting my expertise are key to informed decision-making. Although in the first episode of psychosis I was so overwhelmed by the distressing experiences, that I was less able to be involved in making shared choices about my care, but this does not mean that I was too unwell to have care processes explained to me and understood by me. Despite the experiences I had, there are many enablers and barriers which both promote and obstruct the implementation of SDM; these issues are further highlighted in the next part of the discussion.

Clinicians may be more likely to implement SDM in certain circumstances such as when encounters are initiated by the service user (28). Moreover, service users who participate positively in SDM and who do not dispute their diagnosis, do not reject relevant clinical facts about their diagnosis or treatment, and are not experiencing negative emotional symptoms may be more likely to be invited by clinicians to share in decision-making about their care (24, 28). Additionally, when a service user has capacity, a clinician can facilitate current and future SDM by recording the service user's preferences, values, and health experiences (e.g., hospitalisations and treatments) (6). By using SDM, professionals could help service users to clarify their preferred care plan for future care during acute episodes. In parallel, professionals can learn from service users not only what their preferences are, but also the underlying reasons for these choices; learning effectively from their expressed options.

However, a service user's perceived lack of insight into their mental health condition (10) and safe-guarding concerns about their situation may be a barrier to professionals' willingness to implement SDM (18) because risk management is at the center of mental health practice (17). Moreover, challenges to implementing SDM have been identified when clients are in severe mental distress or lack insight (10), as experienced in my first situation of acute distress. Moreover, professionals sometimes believe that lack of capacity negates the process of SDM as a service user is perceived as unable to participate effectively in decision-making processes (18). However, despite this, many service users with psychiatric conditions retain capacity to make all or most decisions about their own care, even though their capacity can fluctuate. Furthermore, even when a service user lacks capacity, their perspective is still worthy of regard and should be considered (4).

Thus, despite these barriers to the implementation of SDM, input from the service user about their care preferences might provide critical information about how a medication makes them feel or how difficult or easy it is to adhere to specific treatment demands. This therefore suggests in the circumstances such as those described in my first encounter with mental health services, information delivered at the right level and respect for my understanding could have been garnered about my preferences for future treatment and care to support my recovery (5).

In cases where service users are perceived by professionals as lacking insight, capacity, or are assessed as engaging in unsafe behaviors, when the professional believes that the “correct” safe-guarding decision is not agreed in a safety concern, then they may believe they have little option but to reject SDM and to revert to former paternalistic strategies (6); a real anti-thesis to the ideal of recovery (4). For example, clinicians may fear that SDM may lead to non-adherence to medication (17). In such circumstances, professionals may not prioritize and value the reasons why service users choose not to comply with medications which may cause them negative and intolerable adverse effects. In such circumstances, professionals may feel that they are accountable for safety decisions; thus, this highlights that professional responsibilities about the medical and legal limitations of professional accountability need to be clarified (18) and emphasizes the need for value to be placed on listening to and respecting the opinions of the service user, as identified above.

This article has explored the process of SDM through the frame of my autoethnographic account of two experiences of intervention; the former episode in which decision-making was based on paternalistic processes and the latter interaction in which decisions were user-led. This discussion has explored the implementation of SDM and discussed the importance of effective person-centered care in this interaction (30) alongside the importance of a therapeutic alliance (6); which are essential ingredients in the promotion of a recovery serviced promoting agency and empowerment (4).

To support the effective implementation of SDM, clinicians should adhere to the necessary conditions for SDM which are mutual respect and trust, and should provide information in a language understood by service users. There must be attitudinal change in the professional domain, as well in the perspective of some service users, about the place of experiential knowledge in building a therapeutic alliance. Practitioners must, for example, be prepared to listen to treatment options suggested by service users which the professionals themselves have not considered previously, acknowledging the value of experiential wisdom, alongside their own practice wisdom. The implementation of SDM thus has real implications for the place of safe-guarding in mental health care and requires a shift in wider policy to a greater focus on the place of experiential wisdom in decision-making in mental health interventions.

## Data Availability Statement

The original contributions presented in the study are included in the article/supplementary material, further inquiries can be directed to the corresponding author.

## Author Contributions

The author confirms being the sole contributor of this work and has approved it for publication.

## Conflict of Interest

The author declares that the research was conducted in the absence of any commercial or financial relationships that could be construed as a potential conflict of interest.
